# The Laborer Worthy of His Hire

**Published:** 1875-01

**Authors:** 


### Editorial.

---

The Laborer is Worthy of His Hire.—This principle of ethics and right between man and man is older, far older than the inspired book which gives it utterance with divine sanction; it is as old as the race of man; it is ingrained into his very being, part and parcel of himself. Why it is that the law of the land, and to a great extent the public sentiment of the community, should find in the medical profession an exception to the operation of this great natural law of labor and reward, is not easily explainable upon any principle of equity or justice.

Under the existing statute law of Georgia, certain privileged classes are protected in the assertion of their just claims for remuneration, whilst the poor doctor is left at the mercy of his debtor, who may pay or not, as his caprice may lead him. The grocer may furnish his goods, and the mechanic his labor, or either may refuse at his option; but woe be to the poor, starving doctor, if he refuse to render his service, by day or by night, in sunshine or in storm, in summer's sweltering heat or the winter's chilling blast. The grocer furnishes the bon-bons and sweetmeats for the Christmas dinner which unhinges the vital machinery, and then summons the debtor to render at the bar of justice the uttermost farthing; the poor doctor watches through the weary hours of the midnight, disgorging the overloaded stomach and restoring the debtor to health, but to find, when his labor is done, the gates of justice barred against him. The mechanic repairs the doctor's dwelling, and turns his wife and little ones, houseless and homeless, into the world, for his pay; the doctor, by his self-sacrificing care and labor, snatches the mechanic from the jaws of death, and then, with hat in hand, obsequiously pleads for a pittance to feed his famishing household. Such is justice in the Empire State of the South! Strangely enough, under the panoply of "Wisdom, Justice and Moderation," the only means by which the poor doctor can insure his payment out of the goods of his patient is by making sure that he dies!! For, in the latter event, his claim takes precedence; whilst if his conscience, his self-respect, his humanity lead him to diligent and successful endeavors, he is requited with the cold shoulder of the law.

We learn that an effort will be made, at the approaching session of the Georgia Legislature, to secure a small crumb of justice for our patient, long suffering, little complaining and much abused profession. We sincerely trust that the little crumb may be granted.

